# Investing in new technology: the PET experience

**DOI:** 10.1038/sj.bjc.6601109

**Published:** 2003-07-15

**Authors:** I Bradbury, K Facey, G Laking, P Sharp

**Affiliations:** 1 NHS Quality Improvement Scotland, Delta House, 50 West Nile Street, Glasgow G1 2NP, UK; 2 PET Oncology Group, MRC Cyclotron Unit, Hammersmith Hospital, Du Cane Road, London W12 0NN, UK; 3 Bio-Medical Physics & Bioengineering, University of Aberdeen, Foresterhill, Aberdeen AB25 2ZD, UK

## Abstract

As funding available to health services is limited, demonstrating the cost effectiveness of new technologies is an important step in their introduction. This is especially challenging for diagnostic technologies, for which the usual paradigm of reliance on well-designed, randomised controlled trials may not be appropriate. A recently completed Health Technology Assessment of fluorine-18 deoxyglucose (FDG)-positron emission tomography (PET) imaging in cancer management, focused on addressing whether the introduction of PET imaging would be beneficial to patients in the NHS in Scotland, exemplifies some of the challenges. Although a substantial body of published literature exists for this technology, most of the studies report diagnostic accuracy rather than improved patient outcomes. Direct evidence that such improvement occurs, and is sufficient to meet the NHS' criteria for cost effectiveness, from well-designed trials would constitute the most immediately persuasive case for the introduction of PET imaging. In the absence of such evidence, collaboration is required between a variety of disciplines to synthesise evidence from a variety of sources to create informative decision models that estimate long-term patient outcomes. The use of such decision models enabled the Health Technology Board for Scotland (now a part of NHS Quality Improvement Scotland) to determine that FDG-PET imaging is likely to be cost effective in selected applications and identified long-term patient benefits that it would not be possible to study in a randomised controlled trial.

Any health technology that is new or not readily available is likely to generate pressure for immediate implementation by clinical enthusiasts. When, as is the case with positron emission tomography (PET) imaging, the UK is perceived as ‘lagging behind’, the pressure for investment is likely to be particularly intense. However, in any health-care system with limited resources, priorities for investment must be set on the basis of clear evidence of benefit to patients and good value for the money spent.

PET imaging using fluorine-18 deoxyglucose (FDG) has, since its introduction, offered oncologists the possibility of gaining insights into the biochemical activity of tumours in addition to examining the anatomical structure (Phelps, 2000). These insights may be valuable in staging disease, restaging disease after the completion of therapy or detecting tumour response early during therapy.

However, many exciting and potentially valuable therapies compete for funding available to health services – simply introducing all of them is impossible. This has encouraged the development of the Health Technology Assessment (HTA) discipline, and the establishment of the National Institute for Clinical Excellence and the Health Technology Board for Scotland (HTBS) – now part of NHS Quality Improvement Scotland. Each organisation advises the NHS in England and Wales, and NHSScotland, respectively, on whether or not new and existing technologies and therapies appear to offer ‘good value’. Although not all the recommendations of these organisations have been universally acclaimed (Saunders and Valle, 2002), there is considerable support for the idea that some such assessment is needed. But what might constitute evidence of good value for a diagnostic technique such as PET?

In October 2002, HTBS launched its assessment of PET imaging in cancer management (Bradbury *et al*, 2002) – the 13th assessment of PET published since 1990. Why such apparent continuing controversy about a diagnostic technique that was first described in 1958 (Anger, 1958) and whose application in cancer is the subject of over 1800 published articles? Is this, as Ell (2002) suggests, because ‘well-meaning’ people set barriers that are effectively insurmountable, or because there is a genuine mismatch between what is published and what is needed to demonstrate that PET imaging is ‘good value’ for the tax or insurance payer's penny?

These questions can be answered by considering the issues that confronted our own HTA. These issues are relevant not only to PET imaging but also to similar emerging diagnostic technologies, such as microarray genetic assays of cancer (Alizadeh *et al*, 2000; Bittner *et al*, 2000).

The fundamental concept of HTA is that one needs to find evidence that the use of PET imaging can improve the length or quality of patients' lives at reasonable cost, or that it can reduce the overall expenditure without substantially reducing the effectiveness of patient care, that is the ‘higher order’ outcomes in the hierarchy of forms of evidence described by Fryback and Thornbury (1991). Unfortunately, most research published to date has focused on the diagnostic accuracy of PET, with little attention paid to demonstrating the consequences (if any) of improved accuracy for long-term patient outcomes (Lassen, 2001). This has led to the current and rather frustrating situation of enthusiasts declaring that PET imaging is now essential, for example, in staging potentially operable non-small-cell lung cancer (NSCLC) (Medicare, 2001; Reske *et al*, 1996), while a succession of HTA agencies declare that PET staging is ‘interesting’, and that it may be clinically effective but more research is needed (Lassen, 2001; Medical Services Advisory Committee, 2001).

Following the same successful paradigm as in drug applications, HTA agencies tend to call for more prospective randomised studies, in which PET imaging is considered as an alternative or supplement to conventional diagnostic procedures. Patients are treated on the basis of these results following an agreed protocol, and outcomes such as survival, quality of life and cost are recorded. This is all very straightforward, so why have not lots of these studies been undertaken? Indeed, why has only one trial approximating to this recipe for PET been published to date (van Tinteren *et al*, 2002)?

We believe that there are a number of factors responsible for this. Firstly, although such trials would provide ideal evidence of long-term patient benefit, they would be inefficient and possibly unhelpful in assessing the diagnostic accuracy (in a new indication) or utility (when accuracy has already been shown) of PET imaging. This is because the effect of imaging would be confounded with the effects of therapy. Indeed, in many cases, such an approach might be seen as ‘using a poor therapy to judge a good diagnostic method’. Secondly, such trials rely on investigators closely following a protocol that specifies the order in which tests are done, and how the test results will be used to determine treatment. This may happen more rarely than one might wish. For example, one major difficulty in assessing the utility of PET imaging for NHSScotland has been the lack of agreement between oncologists as to how it may best be used. However, the introduction of Managed Clinical Networks is likely to improve this situation (Scottish Executive Health Department, 2001).

Other problems in undertaking trials include:

- the lack of incentive for support from manufacturers, since device authorisation is based mainly on safety rather than efficacy;
- the belief that the case for PET imaging is already proven;
- the lack of incentive for major US centres to undertake such studies, since reimbursement is already available on the basis of accuracy data and some evidence of cost savings (in the high-cost US environment);
- the daunting timescales required for many potentially important studies. For example, studies of delayed or avoided radiotherapy in non-Hodgkin's lymphoma could require a follow-up of 20 years to detect potential second tumours;
- the wide variety of radiotracers potentially available for PET imaging in oncology.

## NSCLC – RANDOMISED CONTROLLED TRIALS

The one application, in which a randomised controlled trial (RCT) has been published involves testing PET in addition to current staging *vs* current methods alone to evaluate NSCLC patients for surgery (van Tinteren *et al*, 2002). This study reported that PET imaging allowed futile (i.e., demonstrably noncurative) thoracotomies to be avoided and was cost saving in the Dutch health-care service. Unfortunately, although it appears reasonable that the avoidance of such surgery may improve the quality of patients' lives, this study did not collect the evidence needed to support this. Since there is no evidence that avoiding unnecessary surgery significantly improves survival, the justification for PET in this case depends ultimately on costs. Since costs may be different in the UK from those in the Netherlands, it is entirely possible that the same study could justify a different decision here, specifically if surgery is substantially cheaper in the UK than in the Netherlands.

Adding to the possibilities for confusion is an Australian RCT, published only as an American Society of Clinical Oncology presentation (Boyer *et al*, 2001), which resulted in no evidence for any difference in futile thoracotomies between the two groups. It appears that some of the discrepancies between these studies are caused by different approaches to treating patients with computed tomography (CT)-positive PET-negative disease in the mediastinum. The Dutch investigators regarded all patients with this stage of disease as inoperable, whereas the Australians would often still operate. Unfortunately, it seems that the trials do not offer definitive conclusions, and that assessment is dependent on decision models.

## NSCLC – MODELLING

Briefly, a decision model attempts to reflect local surgical decision-making, cost structures and the impacts of both treatment and disease on quality of life, and to link the increased accuracy of PET to final outcomes through a decision-tree approach (Dietlein *et al*, 2000). The overall expected benefit from a number of alternative actions may then be compared, and that yielding the highest expected benefit selected as the most cost-effective strategy.

A number of such models of varying sophistication have been published for NSCLC (Gambhir *et al*, 1996; Scott *et al*, 1998; Dietlein *et al*, 2000). We chose to base our assessment of PET in early stage NSCLC on the Dietlein *et al* study, and amended it to take account of recently published accuracy data, costs within NHSScotland and local clinical opinion on how PET imaging would most likely be used. The conclusions from this model are detailed in the published report (Bradbury *et al*, 2002). In summary, the model indicated that PET imaging appears to be cost effective only in patients with no evidence of abnormal lymph nodes on CT scan. All such patients who also appear normal on PET scan should be operated upon. Apparent abnormalities on PET scan should also be confirmed by mediastinoscopy-guided biopsy before denying a patient an operation.

However, the simple single-variable sensitivity analyses we performed suggest that the model conclusions are extremely sensitive to a number of uncertain values, on which more information is required, specifically:

- the quality and length of survival after surgical or nonsurgical treatment for patients with N2 disease;
- the appropriate quality of life ‘penalty’ associated with a thoracotomy (in particular, whether a larger penalty is appropriate for futile thoracotomies);
- the accuracy of mediastinoscopy-guided biopsy;
- the accuracy of PET imaging in detecting distant metastases;
- the actual cost of surgery to NHSScotland.

Note that the accuracy of PET imaging for detecting NSCLC is not part of this list, and that the value of further studies of accuracy in staging mediastinal disease in NSCLC is likely to be minimal, although well-designed studies to assess accuracy in detecting distant metastases should be welcomed. To obtain the required information, a randomised trial of PET imaging is *not* necessarily the only appropriate tool. Many of the data needed may be collected more efficiently from audits of routine practice, or by appropriately designed surveys.

## GUIDING STUDY DESIGN

So, we have moved on somewhat from the blanket approach ‘do more research, do another RCT’, but is it possible to be more precise and to describe how much further research might be needed? The answer is ‘yes’, but with considerable caution, using the emerging tools of probabilistic sensitivity analysis and value of information analysis (Fenwick *et al*, 2000; Laking *et al*, 2002a).

Such methods, developed within the Bayesian decision theory (Berry, 1995), allow one to estimate the value of further information, for example, as acquired from an RCT, in reducing the uncertainty about a possible decision. These methods have been applied successfully in a number of areas to include engineering fields such as hydrology (Reichard and Evans, 1989) and radiation safety (French and Smith, 1997), and in investment appraisal (J Craig, personal communication, February 2003) and have recently been suggested as useful alternatives to the simple statement ‘more research is needed’ (Claxton and Posnett, 1996; Phillips, 2001; Laking *et al*, 2002b).

In outline, the methods are a simple extension of decision modelling. Since our knowledge of the effects of the actions modelled is unlikely to be perfect, there will remain uncertainty. Essentially, the magnitude of this uncertainty will determine how much effort or money should be expended to reduce it.

Of course, if it were all so simple, we would have been doing it for a long time. Setting aside the nontrivial technical challenges, the main barrier to these methods is that to provide sensible answers, one has to take account of *all* the currently available information. Although this may be relatively straightforward for some therapies in which a large body of ‘high-quality’ randomised trial evidence is available, the implications in areas such as diagnostic testing, where trial quality is more variable, and for epidemiological inputs, such as disease prevalence, are substantial and not fully worked out as yet (see, for example, Ades and Cliffe, 2002). Certainly, it will no longer be enough simply to summarise the RCTs, and discard the rest.

## HODGKIN'S DISEASE – MODELLING

As an illustration of this approach, we attempted to assess the net benefit based on a complex (37-state) model of using PET imaging in patients with ‘bulky’ Hodgkin's disease after initial ABVD (doxorubicin, bleomycin, vinblastine and dacarbazine) chemotherapy to determine whether or not the patient should receive immediate consolidative radiotherapy. The model incorporated evidence from all available published studies, both on PET and CT accuracy and on the long-term prognosis of Hodgkin's disease. We compared a ‘current practice’ model of basing the decision on the presence of abnormalities on CT scan to two alternatives, one in which PET imaging was used only in patients with residual CT abnormalities and a second in which PET imaging was used in all the patients regardless of the CT results. It is instructive to consider the wide variety of inputs needed, even for such a relatively simple decision problem.

As the possible adverse effects of radiotherapy are likely to manifest after a number of years (Hancock *et al*, 1993), the model needed to account for the effects not only of initial therapy, but also of potential salvage and palliative therapies. We are grateful to clinical colleagues for advice on the possible pathways for such patients. However, in addition to such ‘structural’ information, the model required the maximum information possible on:

- sensitivity and specificity of PET and CT;
- relapse rates after ABVD therapy, with and without consolidation;
- response rates to salvage therapy;
- the toxicity of salvage therapy;
- survival rates after response to salvage therapy;
- the long-term toxicity of radiotherapy;
- cost data for all the possible therapies.

It is clear that such modelling exercises will not be undertaken lightly and will rely heavily on expert opinion, both to select and to supplement the available data sources. Inevitably, such modelling will be criticised for its possible subjectivity, and will only be plausible if performed within a disciplined framework akin to that provided to the pharmaceutical industry by the International Conference on Harmonization (ICH) guidance (http://www.ifpma.org/ich5e.html) (Facey and Lewis, 1998).

However, we believe that only such complete and necessarily complex modelling exercises will be able to provide both evidence for the cost effectiveness of diagnostic technologies such as PET imaging and appropriate guidance for further research.

For Hodgkin's disease, the modelling results led HTBS to recommend that PET scanning should be used in clinical practice to restage patients after ABVD therapy. In contrast to the implausible 20 years that may be required for a definitive RCT, the modelling exercise occupied 6 months. We recognise, however, the need for more experience on the use of this PET technology in practice, and a key recommendation of the HTA is that treatment and outcome data should be collected on *all* patients undergoing PET imaging.

Interestingly, our model predicts that using PET scanning results to determine therapy in all patients will be more effective than using PET imaging only in CT-positive patients, essentially because the sensitivity of the combined procedure is lower than that of PET or CT imaging alone. We recognise, however, that the policy of imaging only the CT-positive patients is a less dramatic change from current practice, and may therefore be preferred.

## CONCLUSION

The HTA has covered only two of the possible therapeutic areas for FDG-PET imaging in oncology. The process of relating accuracy data to outcomes through careful detailed modelling exercises needs to be extended to other cancers, perhaps most obviously and immediately non-Hodgkin's lymphoma, recurrent head and neck cancer, malignant melanoma and solitary pulmonary nodules. As well as helping to build the evidence base for PET imaging, such large-scale collaboration between economists, statisticians, clinicians and physicists would provide valuable insights into how the process of economic modelling might best be formalised and regulated in HTA. Within NHSScotland, the conclusion of our assessment is best phrased in terms of the classification suggested by Claxton *et al* (2002)*–* ‘implement, but research further’.
